# Effect of nutritional supplementation with lipid-based therapeutic food on body composition of non-severely malnourished African children aged 6–59 months hospitalized with severe pneumonia

**DOI:** 10.1093/tropej/fmaf010

**Published:** 2025-02-22

**Authors:** Damalie Nalwanga, Victor Musiime, Sarah Kiguli, Peter Olupot-Olupot, Florence Alaroker, Robert Opoka, Abner Tagoola, Hellen Mnjala, Christabel Mogaka, Eva Nabawanuka, Elisa Giallongo, Charles Karamagi, André Briend, Kathryn Maitland

**Affiliations:** 1 Department of Paediatrics and Child Health, Makerere University College of Health Sciences, Kampala, Central Region, 256, Uganda; 2 Department of Paediatrics, Makerere University Lung Institute, Kampala, Central Region, 256, Uganda; 3 Research Department, Joint Clinical Research Center, Wakiso, Central Region, 256, Uganda; 4 Department of Paediatrics and Child Health, Mbale Clinical Research Institute, Mbale, Eastern Region, 256, Uganda; 5 Faculty of Health Sciences, Busitema University, Tororo, Eastern Region, 256, Uganda; 6 Soroti Regional Referral Hospital, Soroti, 256, Uganda; 7 Department of Paediatrics and Child Health, The Aga Khan University Hospital Nairobi, Nairobi, Nairobi County, 254, Kenya; 8 Department of Paediatrics, Jinja Regional Referral Hospital, Jinja, 256, Uganda; 9 Research Department, KEMRI-Wellcome Trust Research Programme, Kilifi, 254, Kenya; 10 Statistics, Intensive Care National Audit & Research Centre, London, +44, United Kingdom; 11 Clinical Epidemiology Unit, Department of Internal Medicine, School of Medicine, Makerere University College of Health Sciences, Kampala, Central Region, 256, Uganda; 12 Center for Child, Adolescent and Maternal Health Research, Tampere University Hospital, Tampere University, Tampere, Pirkanmaa, 358, Finland; 13 Faculty of Medicine and Health Technology, Tampere University, Tampere, Pirkanmaa, 358, Finland; 14 Department of Nutrition, Exercise and Sports, University of Copenhagen, Kobenhavn, +45, Denmark; 15 Department of Infectious Disease and Institute of Global Health and Innovation, Imperial College London, London, +44, United Kingdom

**Keywords:** bio-impedance analysis, body composition, children, fat-free-mass fat-mass, malnutrition, pneumonia, ready-to-use therapeutic food

## Abstract

Pneumonia remains an important cause of morbidity and mortality among children in low- and middle-income countries. Poor outcomes are associated with undernutrition. Nutritional supplementation may be beneficial. We examined the effect of supplementation with lipid-based ready-to-use therapeutic food (RUTF) on the body composition of children with severe pneumonia. Non-severely malnourished children (6–59 months) with severe pneumonia enrolled into the Children’s Oxygen Administration Strategies and Nutrition trial in Uganda and Kenya, and randomized to receive a diet supplemented with RUTF (500 Kcal/day) for 56 days versus usual diet alone (control) were included. We assessed arm anthropometry and bioimpedance analysis at admission and days 28, 90, and 180 of follow-up. We used mixed effects linear regression to compare body composition between groups. We included 737 participants (369 in intervention; 368 in control group). The median age was 16 months (IQR; 9, 26), and 58.1% were male. Overall, baseline mean arm fat area (AFA), arm muscle area, and arm muscle circumference were 5.8 ± 1.8 cm^2^, 11.6 ± 2.3 cm^2^, and 12.3 ± 1.2 cm^2^, respectively. The mean fat mass and fat-free mass calculated in 116 participants were 5.5 ± 1.5 kg and 5.5 ± 1.5 kg, respectively. There were modest increases in most body composition parameters. RUTF significantly increased AFA at days 28 and 90 but not at day 180 (*P*-value =.03, .02, and .99, respectively). RUTF did not change other body composition parameters. Despite initial increases in AFA, RUTF did not change the body composition of children with severe pneumonia.

## Introduction

Despite the rollout of effective preventative interventions, pneumonia remains the commonest cause of morbidity and mortality among children under 5 years worldwide, with over 37 million episodes and over 500000 deaths [1]. Over 50% of all-cause mortality in acute illnesses (including pneumonia) in children under 5 is attributed to malnutrition [2, 3]. Severe pneumonia results in a catabolic response, particularly in those with pyrexia and increased work of breathing, resulting in the urgent need to rapidly mobilize energy from fat and amino acids from muscle to assist with the immune response [4–6]. Fat and muscle loss in turn reduces children’s immunity and increases the risk of acquiring more infections, developing severe forms of malnutrition and death [7–9].

Since low- and middle-income countries (LMICs) have a significant number of the world’s undernourished children, it follows that the region has the highest numbers of pneumonia morbidities and mortalities globally [1, 2, 10, 11]. Children in regions with a high prevalence of malnutrition and pneumonia could potentially benefit from nutritional supplementation to restore the fat and muscle mass lost during pneumonia episodes and reduce progression to severe malnutrition and death.

The effectiveness of nutritional support has been demonstrated in a community-based study which showed that nutritional supplementation with a ready-to-use therapeutic food (RUTF) following non-severe illness reduced the incidence of acute malnutrition among 2202 rural Ugandan children aged 6–59 months [12]. On the other hand, the Children’s Oxygen Administration Strategies and Nutrition (COAST-Nutrition ISTCTN10829073) trial in Uganda and Kenya found no benefit of RUTF (in addition to the usual diet) for 56 days on a composite outcome of mortality and Mid-Upper Arm Circumference (MUAC) among 846 children following admission with severe pneumonia compared to usual diet alone [13].

Traditional weight-based anthropometric measures currently used to assess undernutrition among children hospitalized for pneumonia poorly estimate this population's underlying physiologic and functional levels [14, 15]. Body composition is a better method for assessing the effect of nutritional interventions [14, 16]. Currently, there is no relevant data on whether nutritional supplementation with RUTF improves fat and muscle mass in children in LMICs who are recovering from severe pneumonia. This study aimed to investigate the effect of nutritional supplementation with a lipid-based therapeutic food (RUTF) on the body composition of non-severely malnourished children aged 6–59 months hospitalized with severe pneumonia.

## Materials and Methods

### Study design

This is a sub-study of the COAST-Nutrition study which has been described in detail elsewhere [17]. Briefly, the COAST-Nutrition study was a randomized controlled trial involving 846 children hospitalized for severe pneumonia and hypoxemia at 4 referral hospitals in Uganda and Kenya between 2020 and 2022. The primary objective of the COAST-Nutrition study was to establish whether supplementing the usual diet with peanut-based RUTF in children following admission with severe pneumonia will improve survival and MUAC at Day 90 and Day 180.

The study was conducted in accordance with the guidelines in the Declaration of Helsinki and all procedures involving human subjects were approved by the School of Medicine Ethics Research Committee (SOMREC), (2016-030 and amendment 2020-155), in Uganda and KEMRI Scientific and Ethics Review Unit (KEMRI/SERU/CGMRC-C/0053/3300 and amendment C 215/4109) in Kenya. Written informed consent was obtained from eligible participants’ parents/caregivers. The objectives of this analysis were to (i) determine the effect of a nutritional intervention on body composition in children 6–59 months hospitalized for severe pneumonia at days 28, 90, and 180 of follow-up, and (ii) compare body composition between the intervention and control arm over 180 days of follow-up.

### Study population

The COAST-Nutrition study enrolled children 6 months to 12 years admitted to participating sites with severe pneumonia as defined by the World Health Organization (WHO) guidelines [18], and hypoxemia [BitMos pulse oximetry (Bitmos GmbH, Düsseldorf, Germany)] reading of SpO_2_ < 92% recorded in room air over 5 minutes. Children who had severe malnutrition (MUAC < 11.5 cm in children less than 5 years, and/or the presence of bilateral edema), were previously recruited into the trial (to avoid double enrolment), had known chronic lung disease (not including asthma) or had congenital cardiac disease were excluded from the trial.

### Study procedures and follow-up

Baseline demographics, clinical, and body composition data were collected on paper case report forms and entered into a study database. Inpatient care was provided as per the standard protocols. At 48 hours following hospital admission, eligible children were randomized to either supplementary feeding for 56 days (8 weeks) using one 92 g sachet (500 Kcal) of RUTF per day for children < 5 years or to the usual diet alone (control). Children unable to tolerate oral feeds at 48 hours received liquid-based feeds via nasogastric tube until they were able to tolerate oral feeding to start RUTF. Follow-up visits were conducted at discharge, on days 28, 90, and 180 after admission, with a window period around the target date [17].

### Mid-upper arm circumference and triceps skinfold thickness measurement

MUAC was measured using an MUAC tape at the midpoint between the tip of the acromion and the most distal point of the olecranon process on the non-dominant arm, with the arm flexed at a 90° angle. The MUAC was measured twice and the average was recorded to the nearest 0.1 cm [17]. The triceps skin-fold thickness (TST) was measured on the right arm using the Harpenden skinfold calipers^®^ (HaB International Ltd, Southam, United Kingdom) on the posterior surface of the arm midpoint between the tip of the acromion and the most distal point of the olecranon process. Two measurements were taken and if they varied by less than 1 mm, the average was recorded to the nearest 0.2 mm. The measurements were repeated if they varied by more than 1 mm.

### Body composition measurement

We used the two-compartment model which is relatively less complex, less expensive, and less risky to patients to describe body composition. The model divides the body into two compartments: fat-mass (FM) and fat-free mass (FFM)/lean mass which includes muscle, bone, and body organs [19]. We used anthropometry (MUAC and TST) to estimate arm fat and muscle areas using formulae by Rolland-Cachera *et al*. [20] and bioimpedance analysis to measure FFM and FM. These compartment-specific indices could be more sensitive indicators of nutritional status and adiposity [19, 21]. Weight, height, MUAC, and TST were assessed on admission and follow-up using standardized measures [17]. In a subgroup of children enrolled at one site in Uganda, bioimpedance analysis was conducted.

### Estimation of arm fat and muscle

The use of arm anthropometry [arm muscle area (AMA) and arm fat area (AFA)], as proxies for body composition (muscle and fat mass, respectively) in children, is still accepted in communities and for monitoring nutritional interventions because it is cheap and non-invasive [22]. We calculated arm muscle circumference (AMC) from MUAC and TST by the formula: AMC = MUAC – *π* TST. The AMA was calculated with the formula: AMA = (MUAC)^2^/4*π* – MUAC × TST/2 [20]. Rolland-Cachera *et al*. validated the AMA, AFA, and AMC against magnetic resonance imaging and found that these were indeed accurate for the assessment of body composition [20]. Arm fat and muscle area are based on assumptions that: (i) the arm is cylindrical, (ii) subcutaneous fat is evenly distributed around a circular core of muscle, and (iii) TST accurately separates fat and muscle components of the arm and represents twice the thickness of subcutaneous fat in the arm [23]. When validated among healthy and sick children, Chomtho *et al*. found that arm anthropometry and indices calculated from them predict total body and regional FM accurately [22].

### Bioelectrical impedance analysis measurements

Bioimpedance analysis is a non-invasive technique that assesses body composition by measuring the resistance of the body to the flow of small high-frequency electric currents. In this technique, the body is treated as a single cylinder, and measurements are made between the electrodes placed on the wrist and ankle [24]. Bioimpedance measures: Resistance (R), which is the opposition of tissue to the flow of current, Reactance (X_c_), which reflects capacitive losses from cell membranes and Phase Angle (PA), which shows the quality and quantity of soft tissues [25, 26]. From these, the impedance (Z), which represents the opposition of different tissues to the flow of current at 5 and 50 kHz, is estimated [27]. The impedance is used to evaluate total body water, from which FFM/lean mass is estimated. FM is determined by subtracting FFM from body weight [28]. In severely malnourished children, bioimpedance analysis has been used successfully to assess the composition of their weight gain [29], for assessment of body composition during recovery in comparison to non-malnourished controls, and to explore the effect of a reduced RUTF dose on body composition in recovered children [30] among others. It has also been used among children with moderate acute malnutrition to explore the effectiveness of food supplements in increasing fat-free tissue [31]. We measured bioimpedance using the Bodystat^®^ Quadscan 4000 machine (Bodystat Limited, Version 5/12, Isle of Man, British Isles), adhering to the manufacturer’s guidelines at a frequency of 50 kHz. Two bioimpedance assessments were done at each time point. Additionally, we assessed the quality of the participants’ bioimpedance assessments: score 1 for limbs not touching and straight; score 2 for limbs not touching the body; but very slight bends; score 3 for limbs not touching but bent; score 4 for limbs touching; and score 5 for child moving [30]. We excluded participants whose quality assessment in both tests was 4 or 5. Among those with quality 4 or 5 on the first assessment, assessment 2 was considered if the quality score was 1, 2, or 3. FFM was estimated based on the equation below used by the Bodystat^®^ Quadscan 4000 machine:

FFM = 0.61 resistance index (RI) + 0.25 body weight + 1.31: where RI height in cm squared/resistance. The equation has a Standard Error of Estimate of 2.1 kg and an adjusted *R*^2^ of 0.95 [32].

FM was calculated by subtracting FFM from the weight [Weight (kg)] – FFM). FFM and fat mass indices were calculated by dividing FFM and FM by height squared [FFM/Height (m)^2^ and FM/Height (m)^2^], respectively [30].

### Statistical methods

#### Sample size estimation

**Figure 1:**
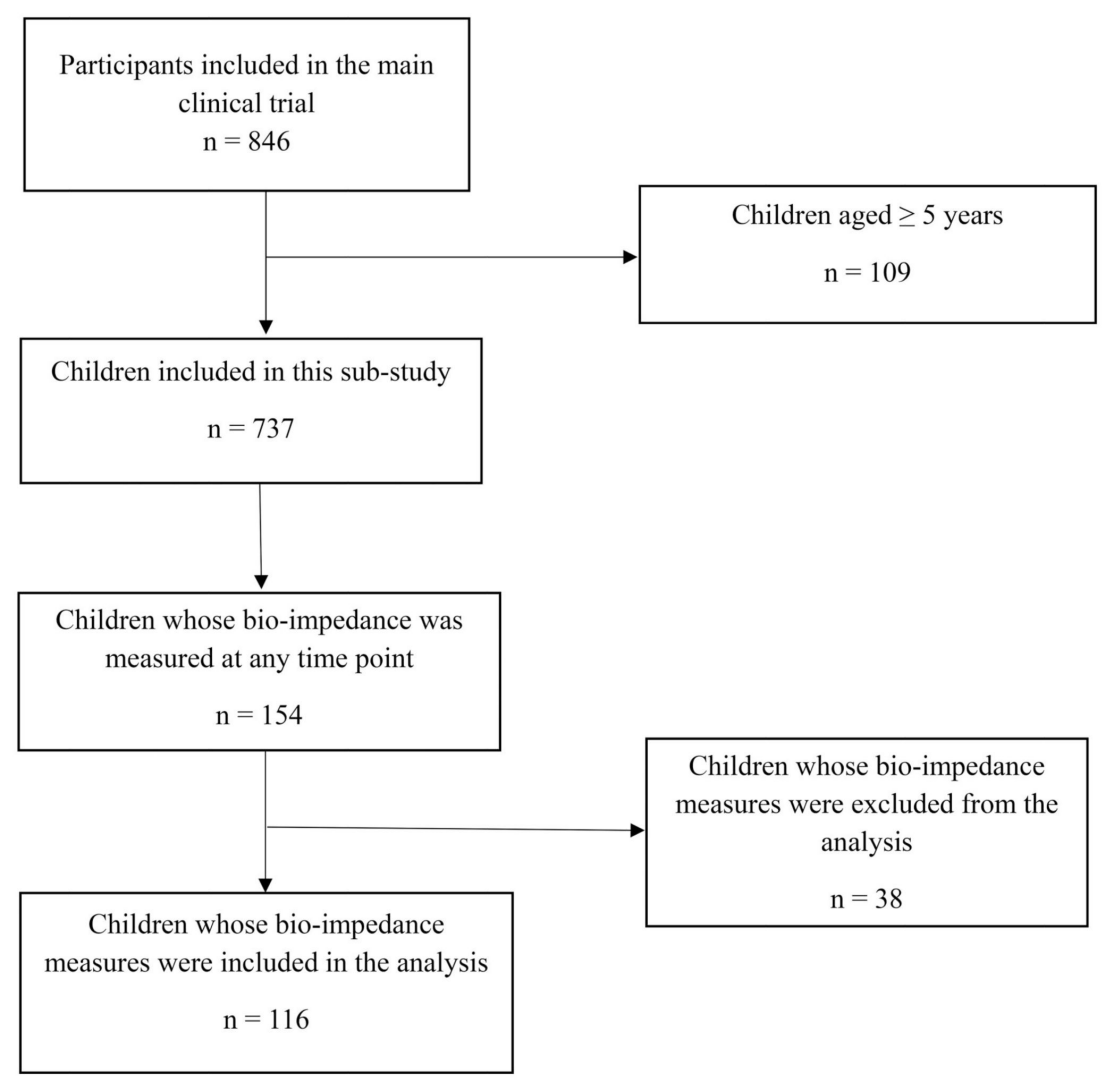
Study flow showing the selection of children aged 6–59 months hospitalized for pneumonia in Uganda and Kenya between 2020 and 2022 included in this sub-study.

The participants included in this study were a convenient sample from a larger clinical trial, the COAST-Nutrition trial. We specifically included children from the main trial who were aged 6–59 months (Fig. 1). We estimated that a mean difference in AFA between the intervention and control arm of 0.3 at day 180, a standard deviation of 2.0, and a sample size of 736 (368 in each group) gives a reasonable confidence interval width of 0.58 for a confidence level of 0.95 [33].

#### Analysis

Before analysis, data were checked for consistency and completeness and summarized. We considered non-overlapping visit windows, and measurements closest to the study visit for the analysis. We used mixed effects linear regression, adjusting for baseline measures of each parameter with a random effect for the health facility at which participants were recruited to investigate the impact of RUTF on body composition at days 28, 90, and 180. We interpreted coefficients and confidence intervals obtained from the mixed effects models. We plotted margins plots to show the change in body composition measures by randomization arm over the 180 days of follow-up. We also plotted spaghetti plots that show each participant’s body composition trajectory over 180 days of follow-up. We used Stata (Version 16 released in June 2019, StataCorp, College Station, Texas, USA) for the analysis [34].

## Results

Overall, a total of 737 participants were included in this study: 369 in the intervention and 368 in the control group. The median age overall was 16 months (IQR 9, 26), and 270 (36.7%) of the participants were aged 6–23 months. Fifty-eight percent (58.1%) of the participants were male, with the majority having been born at term and breastfed beyond 3 months (696, 95.3% and 675, 91.7%), respectively. Routine immunizations were up-to-date in the majority of the study participants (89.9%), and 65.5% had WHO-defined hypoxemia with oxygen saturation <90% in room air. Overall, the mean arm-fat-area, arm-muscle-area, and arm-musclecircumference were 5.8 ± 1.8 cm^2^, 11.6 ± 2.3 cm^2^, and 12.3 ± 1.2 cm^2^, respectively. A subgroup of 116 participants had bioimpedance results analyzed. Of these, the mean FM was 5.5 ± 1.5, and the mean FFM was 5.5 ± 1.5 kg, Table 1.

**Figure 2:**
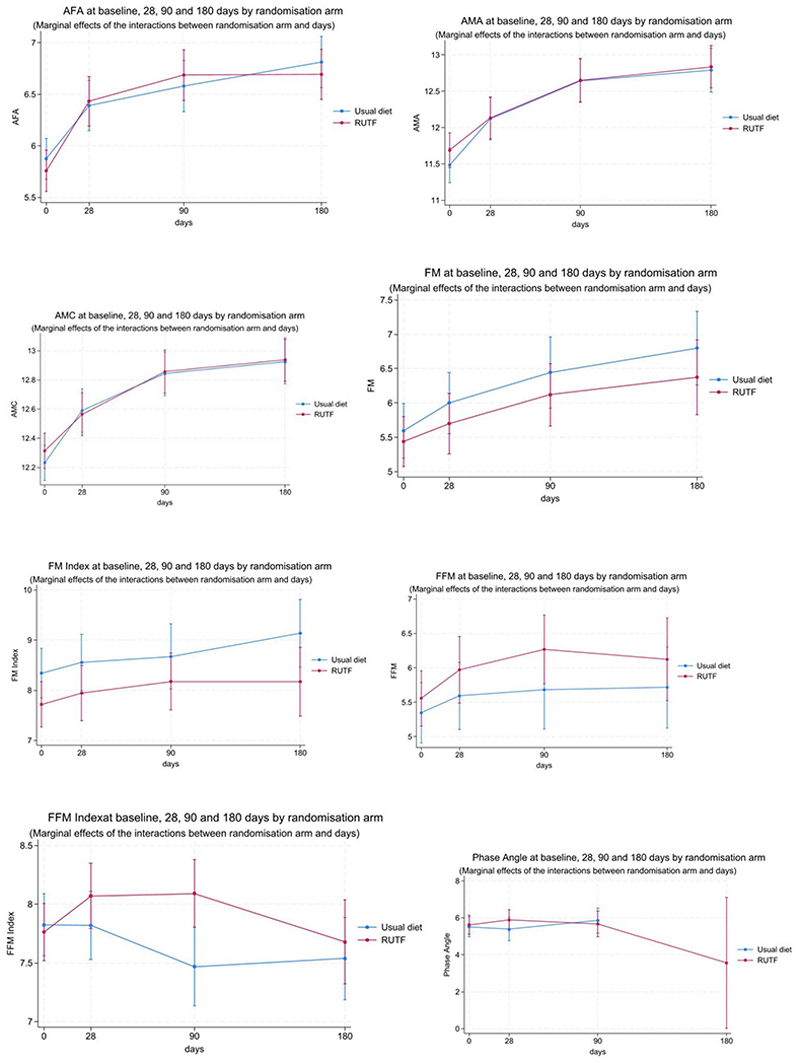
Margin plots of body composition measures for children aged 6-59 months hospitalized for pneumonia in Uganda and Kenya between 2020 and 2022 in the intervention and control arm over 180 days of follow-up.

Follow-up body composition was available for 496 participants at day 28 (251 in the intervention vs 245 in control), 477 at day 90 (241 in the intervention vs 236 in control), and 479 at day 180 (245 in the intervention vs 234 in control). In the bioimpedance analysis subgroup, follow-up results were available for 85 participants at day 28 (43 in the intervention vs 42 in control), 73 at day 90 (42 in the intervention vs 31 in the control), and 57 at day 180 (29 in the intervention vs 28 in control). Overall, there were modest increases in most body composition measures over 180 days of follow-up. The exceptions were FFM and FFM index, Table 2 and Fig. 2. From baseline to day 90, the FFM increased, then reduced slightly by day 180 (intervention arm) and plateaued in the control arm. FFM index slightly increased from baseline, peaking at day 28, then reduced by day 90 and day 180 in the intervention arm, while plateauing in the control arm (Table 2, Fig. 2). The body composition parameters at days 28, 90, and 180 of follow-up are summarized in Table 2, and margin plots of the body composition parameters are presented in Fig. 2. Individual participants’ body composition trajectories over the 180 days of follow-up are shown as spaghetti plots in Fig. S1.

There was no significant difference in body composition parameters between intervention and control arms except the AFA at days 28 and 90 of follow-up on mixed-effects linear regression analysis, Table 3. The intervention arm had a significantly higher mean AFA compared to the control arm as reflected by the coefficient for days 28 (0.18, 95%CI; 0.02–0.35, *P*-value = .03) and 90 (0.25, 95%CI; 0.04–0.45, *P*-value = .02 at day 90). By day 180, there was no difference in AFA between intervention and control arms.

## Discussion

This study aimed to investigate the potential effect of nutritional supplementation with a lipid-based therapeutic food (RUTF) on the body composition of non-severely malnourished children aged 6–59 months hospitalized with severe pneumonia in LMICs. We showed that a diet supplemented with RUTF given over 8 weeks increased AFA by 18% and 24% at days 28 and 90, respectively. By day 180, there was no significant difference in AFA between the two groups. There was no significant effect on other parameters, including AMC, AMA, FM, FMI, FFM, FFMI, or PA compared to the usual diet only.

An initial increase in AFA in the intervention arm could be explained by the high energy intake after a period of reduced energy intake as a consequence of severe illness. Dulloo *et al*. found that higher body fat before a period of semi-starvation resulted in higher fat deposits during refeeding [35]. In general, the studied population was predominantly well-nourished with good baseline AFA, so this might explain the initial increase. However, there was no difference in fat mass estimated from bioimpedance between arms, possibly due to the limited sample size of the subgroup of participants. The observed differences were modest and may not be clinically relevant as the parent trial showed that RUTF did not significantly reduce mortality or change MUAC [36]. The modest fat gain that did not persist to day 180 is also reassuring considering the concerns of increased adiposity associated with lipid-based nutritional supplements like RUTF [37].

A RUTF-supplemented diet did not significantly affect muscle mass indices (AMA and AMC) in the intervention versus control arm. Studies supplementing children with a similar dose of RUTF showed that a higher proportion of weight gain during supplementation was FFM [30, 31]. In contrast to our study, these studies lacked a control group for comparison, and supplemented children for longer (12 weeks compared to 8 weeks in this study). The studies were also conducted among severely and moderately malnourished children, while our population was predominantly well-nourished. There are limited data comparing body composition in intervention (RUTF) versus control among predominantly well-nourished children with severe pneumonia in LMICs. The effect of dose, treatment duration, and alternative nutrient proportions on FM and FFM were outside the scope of this study. However, protein-based food may be more beneficial to increase muscle mass, which is hypothesized to be critical for survival in this population as respiratory muscles have high energy requirement [38]. A predominantly undernourished population may also benefit from nutritional supplementation.

### Strengths and limitations

To our knowledge, this is the first study to explore the effect of RUTF on the body composition of children hospitalized for severe pneumonia. The study was nested in a randomized controlled trial which minimized bias. However, there are several limitations. Missing data attributed to mortality, missed visits, and loss to follow-up may have caused a loss of power. The practical limitations of bioimpedance analysis in smaller children, who are unable to keep still and thus cause recording artifacts, resulted in the exclusion of some measurements from the analysis. We expect an imbalance of baseline characteristics between those surviving through follow-up and those who died or were lost to follow-up as lower body composition is associated with higher mortality [39]. We estimated body composition using arm anthropometry and bioimpedance analysis which inherently have limitations related to formulae assumptions and validation of utilized equations, respectively [20, 32]. Additionally, they are not the gold standard for assessing body composition. However, the methods we used to assess arm anthropometry and bio-impedance analysis in this study are practical, feasible, and reasonably cheap for implementation in LMICs.

## Conclusions

Despite significant increases in AFA at days 28 and 90, a lipid-based RUTF-supplemented diet did not significantly change the body composition of non-severely malnourished children following hospitalization with severe pneumonia compared to children on usual diet alone.

## Figures and Tables

**Table 1:** Description of baseline characteristics of children aged 6–59 months hospitalized for pneumonia in Uganda and Kenya between 2020 and 2022

Parameter	Overall *(n =* 737)	Intervention (*n* = 369)	Control (*n* = 368)
Age (months); Median (IQR)	16 (9, 26)	17 (9, 30)	16 (8, 24)
Age category: 6–11 months	253 (34.4%)	128 (34.7%)	125 (34%)
12–24 months	270 (36.7%)	135 (36.6%)	135 (36.8%)
25–59 months	214 (28.9%)	106 (28.7%)	108 (29.2%)
Sex: Male *(n,* %)	428 (58.1%)	210 (56.9%)	218 (59.1%)
Children born at term: Yes (*n*, %)	696 (95.3%)	348 (95.1%)	348 (95.6 %)
Breastfeeding history: Yes (*n*, %)	675 (91.7%)	342 (92.7%)	333 (90.7%)
Immunization UpToDate: Yes (*n*, %)	473 (89.9%)	241 (90.6%)	232 (89.2%)
Chest indrawing; Yes, (*n*, %)	677 (92.0%)	337 (91.6%)	340 (92.4%)
Oxygen saturation < 90%	483 (65.5%)	242 (65.6%)	241 (65.4%)
MUAC; Mean (SD), cm	14.7 (1.3)	14.7 (1.4)	14.7 (1.3)
TST; Mean (SD), mm	7.8 (1.9)	8.0 (1.9)	7.9 (1.9)
HIV status known; Positive, (*n*, %)	6 (0.8%)	2 (0.6%)	4 (1.1%)
AFA, cm^2^: Mean (SD)	5.8 (1.8)	5.7 (1.7)	5.9 (1.8)
AMA, cm^2^: Mean (SD)	11.6 (2.3)	11.7 (2.5)	11.5 (2.2)
AMC, cm^2^: Mean (SD)	12.3 (1.2)	12.3 (1.2)	12.2 (1.1)
FM^a^, kg: Mean (SD)	5.5 (1.5)	5.4 (1.6)	5.6 (1.5)
FMI^a^ kg/m^2^: Mean (SD)	8.0 (2.1)	7.7 (1.9)	8.3 (2.3)
FFM^a^, kg/m^2^: Mean (SD)	5.5 (1.5)	5.6 (1.6)	5.3 (1.4)
FFMI^a^: Mean (SD)	7.8 (1.2)	7.8 (0.9)	7.8 (1.4)
PA^a^	5.6 (1.8)	5.6 (2.0)	5.5 (1.6)

a Sample size; Overall sample size = 116, Intervention sample size = 63, Control sample size = 53; IQR, interquartile range; SD, standard deviation; MUAC, mid-upper arm circumference; TST, triceps skinfold thickness, AFA, arm fat area; AMA, arm muscle area; AMC, arm muscle circumference; FM, fat mass; FMI, fat mass index; FFMI, fat-free mass index.

**Table 2:** Means of body composition parameters of children aged 6–59 months hospitalized for pneumonia in Uganda and Kenya between 2020 and 2022 in the intervention versus control arm at days 28, 90, and 180 of follow-up.

Parameter	Overall, mean (SD)	Intervention, mean (SD)	Control, mean (SD)
**AFA**			
Day 28^a^	6.4 (2.0)	6.4 (2.0)	6.4 (2.0)
Day 90^b^	6.6 (2.1)	6.7 (2.0)	6.6 (2.1)
Day 180^c^	6.8 (2.1)	6.7 (2.0)	6.8 (2.1)
**AMA**			
Day 28^a^	12.1 (2.2)	12.1 (2.4)	12.1 (2.1)
Day 90^b^	12.6 (2.4)	12.6 (2.4)	12.6 (2.4)
Day 180^c^	12.8 (2.4)	12.8 (2.5)	12.8 (2.4)
**AMC**			
Day 28^a^	12.6 (1.2)	12.6 (1.4)	12.6 (1.0)
Day 90^b^	12.8 (1.1)	12.8 (1.1)	12.8 (1.2)
Day 180^c^	12.9 (1.2)	12.9 (1.2)	12.9 (1.2)
**FFM**			
Day 28^d^	5.8 (1.7)	6.0 (1.9)	5.6 (1.4)
Day 90^e^	6.0 (1.7)	6.2 (1.9)	5.7 (1.4)
Day 180^f^	5.9 (1.7)	6.1 (1.9)	5.7 (1.4)
**FFMI**			
Day 28^d^	8.0 (0.8)	8.1 (0.8)	7.8 (0.9)
Day 90^e^	7.8 (1.0)	8.1 (1.2)	7.5 (0.7)
Day 180^f^	7.6 (0.8)	7.7 (0.8)	7.5 (0.8)
**FM**			
Day 28^d^	5.8 (1.3)	5.7 (1.3)	6.0 (1.3)
Day 90^e^	6.3 (1.6)	6.1 (1.7)	6.4 (1.3)
Day 180^f^	6.6 (1.4)	6.4 (1.7)	6.8 (1.2)
**PA**			
Day 28^d^	5.6 (1.8)	5.9 (1.9)	5.4 (1.7)
Day 90^e^	5.8 (1.7)	5.7 (1.6)	5.9 (1.9)
Day 180^g^	–	–	–

Sample size:

a Overall: *n =* 496, Intervention: *n =* 251, Control: *n =* 245.

b Overall: *n =* 477, Intervention: *n =* 241, Control: *n =* 236.

c Overall: *n =* 479, Intervention: *n =* 245, Control: *n =* 234.

d Overall: *n =* 85, Intervention: *n =* 43, Control: *n =* 42.

e Overall: *n =* 73, Intervention: *n =* 42, Control: *n =* 31.

f Overall: *n =* 57, Intervention: *n =* 29 Control: *n =* 28.

g Insufficient observations for analysis.

AFA, arm fat area; AMA, arm muscle area; AMC, arm muscle circumference; FM, fat mass; FMI, fat-free mass index; FFMI, fat-free mass index.

**Table 3:** Mixed effects linear regression analysis of body composition over 180 days of follow-up for children aged 6–59 months hospitalized for pneumonia in Uganda and Kenya between 2020 and 2022 in intervention versus control arms.

Variable	Unadjusted coefficient	95% confidence interval	Adjusted coefficient	95% confidence interval	*P*-value
**AFA**					
Day 28^a^	0.04	−0.31 to −0.39	0.18	0.02–0.35	**.03**
Day 90^b^	0.11	−0.26 to 0.48	0.25	0.04–0.46	**.02**
Day 180^c^	−0.12	−0.49 to 0.25	−0.00	−0.25 to 0.25	.99
**AMA**					
Day 28^a^	0.01	−0.38 to 0.40	−0.14	−0.43 to 0.14	.32
Day 90^b^	0.01	−0.42 to 0.44	−0.14	−0.47 to 0.19	.40
Day 180^c^	0.05	−0.39 to 0.49	−0.09	−0.45 to 0.27	.62
**AMC**					
Day 28^a^	−0.02	−0.24 to 0.19	−0.09	−0.26 to 0.08	.31
Day 90^b^	0.01	−0.19 to 0.22	−0.05	−0.21 to 0.11	.54
Day 180^c^	0.02	−0.20 to −0.23	−0.04	−0.21 to 0.13	.65
**FFM**					
Day 28^d^	0.38	−0.33 to 1.09	0.16	−0.16 to 0.48	.34
Day 90^e^	0.59	−0.22 to −1.40	0.08	−0.23 to 0.39	.63
Day 180^f^	0.41	−0.47 to −1.29	−0.11	−0.37 to 0.15	.41
**FFMI**					
Day 28^d^	0.25	−0.09 to 0.59	0.19	−0.11 to 0.49	.22
Day 90^e^	0.62	0.17-1.07	0.24	−0.05 to 0.54	.11
Day 180^f^	0.14	−0.26 to 0.54	0.05	−0.28 to 0.39	.75
**FM**					
Day 28^d^	−0.30	−0.85 to −0.25	−0.26	−0.67 to 0.15	.22
Day 90^e^	−0.33	−1.07 to 0.42	0.10	−0.41 to 0.60	.71
Day 180^f^	−0.42	−1.19 to 0.34	0.16	−0.39 to 0.72	.56
**FMI**					
Day 28^d^	−0.61	−1.28 to −0.06	−0.38	−0.95 to 0.19	.19
Day 90^e^	−0.49	−1.43 to 0.44	0.16	−0.50 to 0.81	.64
Day 180^f^	−0.97	−1.74 to −0.19	−0.11	−0.76 to 0.55	.75
**PA**					
Day 28^g^	0.49	−0.37 to −1.35	−0.07	−0.92 to 0.79	.88
Day 90^h^	−0.18	−1.12 to 0.75	−0.27	−1.03 to 0.49	.49
Day 180^i^	–		–	–	–

Sample size:

a *n =* 496.

b *n =* 477.

c *n =* 479.

d *n =* 78.

e *n =* 64.

f *n =* 54.

g *n =* 53.

h *n =* 40.

i Insufficient observations for analysis. AFA = arm fat area; AMA = arm muscle area; AMC = arm muscle circumference; FM = fat mass; FMI = fat-free mass index; FFMI = fat-free mass index. Statistically significant differences in bold.

## Data Availability

Data will be made available upon reasonable request to the last author, Prof. Kathryn Maitland.
